# Genome-Scale CRISPR-Cas9 Transcriptional Activation Screening in Metformin Resistance Related Gene of Prostate Cancer

**DOI:** 10.3389/fcell.2020.616332

**Published:** 2021-01-26

**Authors:** Jiahong Chen, Yaqiang Huang, Zhenfeng Tang, Maozhang Li, Xiaohui Ling, Jinxian Liao, Xiaobo Zhou, Shumin Fang, Haibo Zhao, Weide Zhong, Xia Yuan

**Affiliations:** ^1^Department of Urology, Huizhou Municipal Central Hospital, Huizhou, China; ^2^Department of Urology, Zhongshan City People's Hospital, Zhongshan, China; ^3^Guangdong Key Laboratory of Urology, Department of Urology, Minimally Invasive Surgery Center, Guangzhou Urology Research Institute, The First Affiliated Hospital of Guangzhou Medical University, Guangzhou, China; ^4^Reproductive Medicine Centre, Huizhou Central People's Hospital, Guangdong Medical University, Huizhou, China; ^5^Guangdong Provincial Institute of Nephrology, Nanfang Hospital, Southern Medical University, Guangzhou, China; ^6^Department of Urology, The Fifth Affiliated Hospital of Guangzhou Medical University, Guangzhou, China; ^7^Guangdong Key Laboratory of Clinical Molecular Medicine and Diagnostics, Department of Urology, Guangzhou First People's Hospital, School of Medicine, South China University of Technology, Guangzhou, China

**Keywords:** prostate cancer, CRISPR, metformin, RAD9A, whole-genome, tumor immune microenvironment

## Abstract

Metformin is a classic type II diabetes drug which possesses anti-tumor properties for various cancers. However, different cancers do not respond to metformin with the same effectiveness or acquire resistance. Thus, searching for vulnerabilities of metformin-resistant prostate cancer is a promising strategy to improve the therapeutic efficiency of the drug. A genome-scale CRISPR-Cas9 activation library search targeting 23,430 genes was conducted to identify the genes that confer resistance to metformin in prostate cancer cells. Candidate genes were selected by total reads of sgRNA and sgRNA diversity, and then a CCK8 assay was used to verify their resistance to metformin. Interestingly, we discovered that the activation of ECE1, ABCA12, BPY2, EEF1A1, RAD9A, and NIPSNAP1 contributed to *in vitro* resistance to metformin in DU145 and PC3 cell lines. Notably, a high level of RAD9A, with poor prognosis in PCa, was the most significant gene in the CCK8 assay. Furthermore, we discerned the tumor immune microenvironment with RAD9A expression by CIBERSORT. These results suggested that a high level of RAD9A may upregulate regulatory T cells to counterbalance metformin in the tumor immune microenvironment.

## Introduction

Despite the progress made in screening, diagnosis, and therapy for prostate cancer (PCa), there are still more than one million new cases and 300,000 related deaths annually worldwide (Ferlay et al., 2013, 2015). Based on the diagnostic tools and therapeutic strategies of PCa, significant advances in the diagnosis and treatment of PCa have been made (Cuccurullo et al., 2017; Fanti et al., 2018). The androgen/androgen receptor (AR) axis plays a vital role in the pathogenesis of PCa, thereby androgen deprivation therapy (ADT) remains the first choice for this disease (Di Zazzo et al., 2019). New kinds of androgen inhibitors or androgen receptor (AR) antagonists, such as enzalutamide, have improved the survival percentage of PCa patients (Attard and Antonarakis, 2016; Baciarello et al., 2018). Despite the initial response, PCa usually develops resistance to these drugs and progresses to castration-resistant prostate cancer (CRPC) stages. Thus, preventing progression is a major concern for patients with drug resistance after surgery.

Metformin is a classic type II diabetes drug which may possess anti-tumor properties for various cancers, such as colon cancer (Boorjian et al., 2012), pancreatic cancer (Duan et al., 2017), breast cancer (Wahdan-Alaswad et al., 2016), and prostate cancer (Gong et al., 2006; Comstock et al., 2007; Tong et al., 2017). Metformin mainly blocks hepatic gluconeogenesis and anti-tumor cells by modulating glucose metabolism due to the cancer cells' frequent utilization of the Warburg effect to generate ATP (Akula et al., 2019). Accumulating evidence implies that Metformin inhibits cancer proliferation by activating the AMPK pathway, which suppresses the mammalian target of the rapamycin (mTOR) pathway, inducing apoptosis and reducing proliferation (Han et al., 2015; Zhao et al., 2015). In addition, metformin has multiple antineoplastic effects (Gonzalez-Angulo and Meric-Bernstam, 2010; Cameron et al., 2016; Heckman-Stoddard et al., 2017) through the intervention of the IGF-1 signaling pathway, the inhibition of the AR pathway, and the modulation of the immune response. However, not all types of cancer respond to metformin with the same effectiveness or acquire resistance (Bansal et al., 2015; Scherbakov et al., 2016). Thus, more investigations into the potential roles of metformin and its anti-cancer effects on PCa could reveal a novel strategy that is safe and economical in treating PCa.

The CRISPR-Cas9 system and the progress of high-throughput sequencing (htseq) techniques have given us the possibility to conduct genome-wide screening in mammalian cells (Shalem et al., 2014; Wang et al., 2014; Bester et al., 2018). In the drug resistance field, many new novel targets were discovered. A genome-scale CRISPR-Cas9 knockout screening (Cao et al., 2018) found nine genes participating in imatinib-resistant cells. Another group found that MSH2 took part in cisplatin resistance in bladder cancer cell lines by performing a whole-genome CRISPR screening. Huang et al. identified that NF-κB/E2F6 was responsible for temozolomide resistance in glioblastoma using the CRISPR-Cas9 genome-wide screening system (Huang et al., 2019). Detecting for vulnerabilities of metformin insensitivity in PCa is a promising way to improve the therapeutic efficiency of metformin. In this study, we took a novel approach to find genes of metformin insensitivity by performing the first genome-wide CRISPRa screening of metformin resistance in PCa.

## Materials and Methods

All experiments involving human tissues were approved by the Ethics Committee of The First Affiliated Hospital of Guangzhou Medical University, PR of China.

### Cell Culture

The prostate cancer cells of human DU145 and PC3 were purchased from the American Type Culture Collection (ATCC, USA) and cultured in DMEM (HyClone, SH30022.01B) medium containing 10% fetal bovine serum (FBS, Gibco) and 100 U/mL of double antibiotics (penicillin and streptomycin, TBD, PS2004HY) in a humidified incubator with 5% CO_2_ at 37°C.

### Cytotoxicity of Metformin *in vitro*

To find out the minimum lethal dose (MLD) of metformin, DU145 cells were treated with 0, 50, 100, 150, and 200 mM of metformin and PC3 cells were treated with metformin at 0, 12.5, 50, 100, and 150 mM concentrations for 24 h. The cell morphology was observed by an optical microscope (Nikon) and cell viability was conducted using a cell counting kit-8 (CCK-8 Kit) (Beyotime, Shanghai, China).

### Lentiviral Packaging and Infection

The CRISPR/Cas9 Synergistic Activation Mediator (SAM) pooled library plasmids was acquired from Addgene (https://www.addgene.org/crispr/libraries/). The CRISPR/Cas9 SAM pooled library plasmids, pCMV-VSV-G, pMDLg, pRRE, and pRSV-Rev were added into 100 μL of Opti-MEM in a ratio of 3:3:1:1:1, and then mixed with polyethylenimine (PEI), briefly vortexed and incubated for 15 min. The cell supernatant containing lentiviruses was collected after 48 h. DU145 cells were transduced at a calculated multiplicity of infection (MOI) (Sanjana et al., 2014; Shalem et al., 2014) of 0.4 followed by selection with hygromycin B (YEASEN, 60225ES03) and blasticidin (MDBio, D0120601) for 15 d. A polymerase chain reaction (PCR) was applied to identify the successful transfection of lentiCRISPRa vector in DU145 cells. CRISPRa-F: TCTTGTGGAAAGGACGAAACACCG and CRISPRa-R: CTCCTTTCAAGACCTAGGATC were selected as the PCR amplification regions (209 bp) from the lentiCRISPRa vector, followed by electrophoresis. The thermocycling parameters of PCR were 95°C for 60 s, 30 cycles of (95°C for 10 s, 56°C for 10 s, 72°C for 30 s), and 72°C for 1 min.

### Performing the CRISPRa Resistance Screen

DU145 cells were plated in quadruplicate for each condition. Cells were treated with metformin (100 mM) for 24 h or vehicle for 24 h. After three rounds of metformin treatment, the treatment media were removed and cells were allowed to grow to confluency prior to harvesting the genomic DNA.

### Genomic DNA Extraction and PCR Products

The HiPure Tissue DNA Mini Kit (Magen) was used to extract genomic DNA of metformin-resistant DU145 cells. The sgRNA sequences of each sample were amplified by polymerase chain reaction (PCR) from genomic DNA using primers containing adaptor and barcoding sequences. For PCR, the primers CRISPRa-F (TCTTGTGGAAAGGACGAAACACCG) and CRISPRa-R (CTCCTTTCAAGACCTAGGATC) were used. The thermocycling parameters were: 95°C for 60 s, 30 cycles of (95°C for 10 s, 60°C for 10 s, 72°C for 30 s), and 72°C for 1 min. After the PCR products were electrophoresed, the HiPure Gel Pure DNA Mini Kit was used for gel extraction.

### sgRNA Deep Sequencing and Enrichment Analysis

HiSeq2500 (Illumina Inc., San Diego, CA, USA) was used for sequencing. Htseq (Anders et al., 2015) was used to count the sgRNA. The top resistant genes were identified by ranking the total reads of sgRNA and sgRNA diversity (number of detected different sgRNAs that target to the same gene) (Cao et al., 2018).

### Cytotoxicity of Metformin in Transfected Cells With Candidate Genes

DU145 and PC3 cells were seeded in 6-well plates and transient transfection was performed with a single sgRNA vector. Then the cell viability assay was conducted using cell counting kit-8 (CCK-8 Kit) (Beyotime, Shanghai, China) according to the manufacturer's instructions. Briefly, the cells (8 × 10^3^ cells per well) were cultured in 96-well plates in triplicate. After allowing the cells to attach to the bottom of the plate for 12 h, the cells were then treated with different concentrations of metformin (PC3 50, 80 μM and DU145 80, 100 mM) or DMSO vehicle for 24 h. And then, 10 μL of the CCK-8 solution was added to each well, and the absorbance (450 nm) was measured by a microplate reader (BioTeke).

### Quantitative Reverse Transcriptase PCR

The EEF1A1, BPY2, ABCA12, ECE1, TAF1L, C20orf203, NIPSNAP1, and RAD9A mRNA levels (see Table 1 for primer sequences) were detected by real-time quantitative reverse transcriptase PCR (q-PCR). Total RNA was extracted using the total RNA Rapid Extraction Kit (BIOTEKE, RP1201) according to the manufacturer's instructions. The SYBR Green® Realtime PCR Master Mix (#QPK-201, Toyobo Co, Ltd, Osaka, Japan) was used for quantitative reverse transcriptase PCR assays. The data were analyzed with an Applied Biosystems 7900 Real Time PCR System. Target genes were normalized to the mean β-actin expression.

**Table 1:** Primer sequences for q-PCR.

**Primer name**	**Product size**	**Sequences**
β-actin	185 bp	F: TGGCACCAGCACAATGAA
		R: CTAAGTCATAGTCCGCCTAGAAGCA
RAD9A	114 bp	F: CACCCAAGAAGTTCCGCTCA
		R: TCTTGGTTCAGCCTTCACCC
NIPSNAP1	178 bp	F: CACAAAGTGGATCCCCGGAA
		R: CGAGTGAGCATGGGTAGTCC
EEF1A1	143 bp	F: GAAAGCTGAGCGTGAACGTG
		R: AGTCAGCCTGAGATGTCCCT
BPY2	186 bp	F: ACTTCTGACTATGCCCAGCCT
		R: GCAGCACCTGTGAAAATCTGG
C20orf203	247 bp	F: CTCCAATTCATCACGGTCGCT
		R: GCACAGCCTCGGTCCCTAAT
ECE1	226 bp	F: ACCATCTTCTACCCCGTGGA
		R: GACAGGTCTTCTTGGTCCCG
TAF1L	123 bp	F: AAGAGTAAAGATCGGCCACG
		R: CATCCCTGTGCGTTTGAAGT
ABCA12	155 bp	F: TCTTCCCAGGGACATACGGT
		R: GGCAGATGGGTTGGTGTTCT

### Bioinformatic Analysis of DNA and RNA Level

We compared the mRNA expression of RAD9A between cancer and adjacent normal tissues by GEPIA (Gene Expression Profiling Interactive Analysis) in the TCGA dataset (http://gepia.cancer-pku.cn/). Next, we analyzed the RAD9A alterations and networks in three prostate adenocarcinoma databases (TCGA PanCancer dataset, TCGA Cell 2015 dataset, TCGA Firehose Legacy) by using the cBioPortal (https://www.cbioportal.org/) online tool. Kyoto Encyclopedia of Genes and Genomes (KEGG) pathway analysis was used to explore the potential functions of genes correlated with RAD9A from the TCGA dataset by OmicShare (https://www.omicshare.com/tools/). The gene correlation cutoff was set at 0.3, and the *Q*-value at < 0.01.

### Immunohistochemistry Analysis

The RAD9A protein expression in the TMA (cat no. PR808b, Xi'an Alenabio Biotech Company, Ltd.) was detected by immunohistochemistry and evaluated with the immunoreactivity scores (IRS) system as we previously described (Lin et al., 2017). A primary antibody against RAD9A (abs136198, Absin Bioscience Inc) was used in these studies. All experiments involving human tissues were approved by the Ethics Committee of Huizhou Municipal Central Hospital, China.

### Bioinformatic Analysis of Protein Level

We used GeneMANIA (http://genemania.org) and STRING (https://string-db.org/) which are two web tools for identifying protein and protein interactions.

### Immune Cell Infiltration Analysis

CIBERSORT (Cell-type Identification by Estimating Relative Subsets of RNA Transcripts; http://cibersort.stanford.edu) was used to characterize the infiltration of 22 kinds of immune cell types with the RNA expression profile of each patient A (Newman et al., 2015). We obtained the abundance ratio matrix of 22 immune cell types with the criteria of *P* < 0.05. We also compared the different infiltration levels of 22 immune cell types in low and high RAD9A expression by “ggplot2” packages.

### Statistical Analysis

Our data were expressed as mean ± *SD*. Analyses were performed using the SPSS 20.0 statistical software (SPSS Inc, IL, USA). Comparisons between groups were analyzed through a t test. A *p* < 0.05 was considered statistically significant.

## Results

### Whole-Genome CRISPR Library Screening for Genes Associated With Metformin Resistance

**Figure 1:**
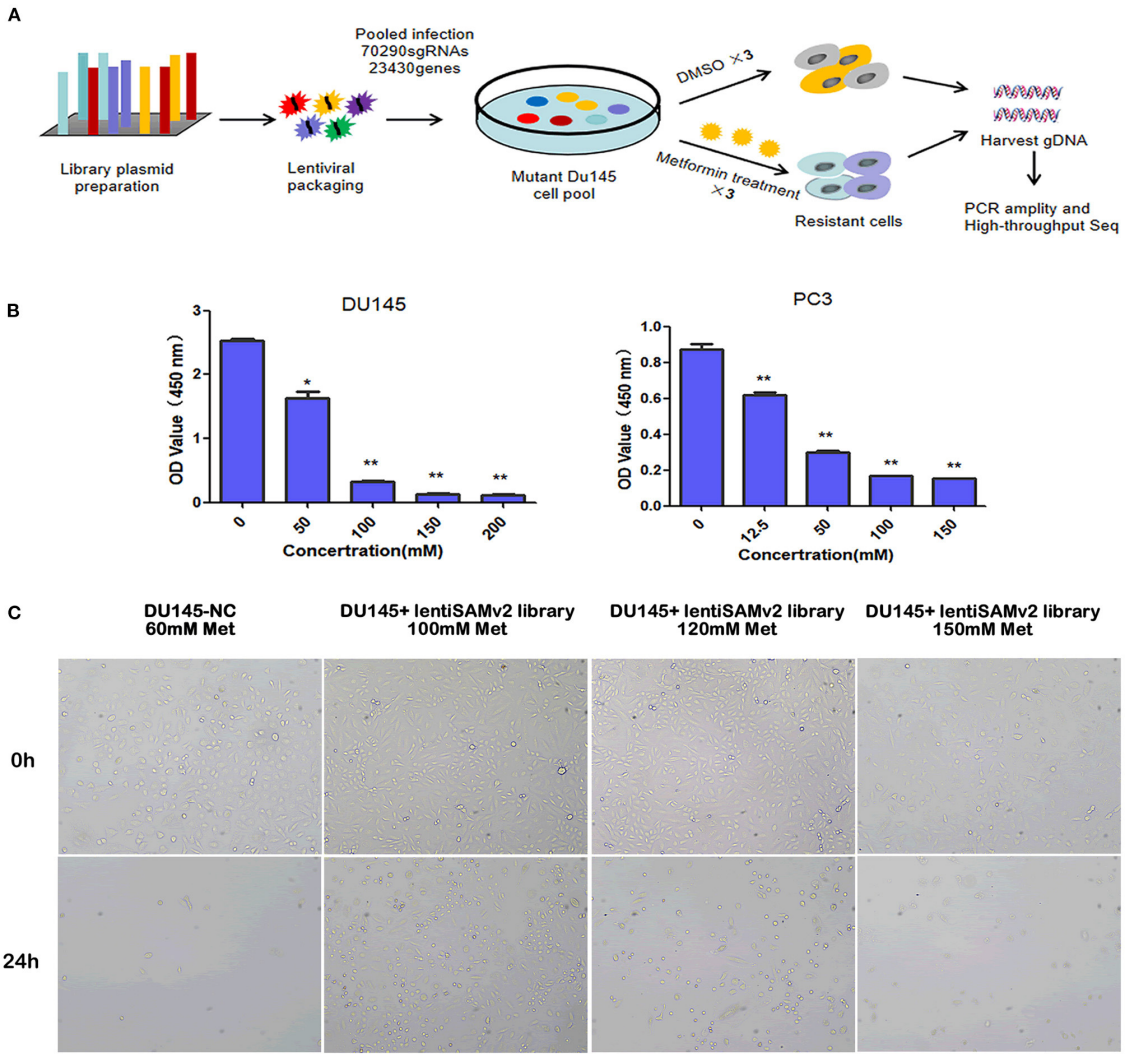
Schematic of functional screening by the CRISPR/Cas9 SAM pooled library and metformin treatment. **(A)** Schematic of metformin-resistant DU145 cells construction for high-throughput sequencing analysis; **(B)** PC3 (0, 12.5, 50, 100, 150 mM) and DU145 (0, 50, 100, 150, 200, 200 mM) cell lines were treated with the indicated doses of metformin for 24 h, and cell viability was measured using a CCK8 assay. Data were represented as mean ± SEM of a representative experiment (of three experiments); **(C)** optical microscopic images of DU145 cells transfected with lentiSAMv2 control or lentiSAMv2 pooled library and treated with metformin (60, 100, 120, 150 mM).

We executed an unbiased gain-of-function screening using the human CRISPR/Cas9 SAM pooled library to identify sgRNA constructs that were enriched in metformin-treated PCa cells (Figure 1A). Supplementary Figure 1 shows the successful transfection of the lentiSAMv2 vector in DU145 cells by electrophoresis, indicating the activation of target genes. To determine the minimum lethal dose (MLD) of metformin, PCa cells were treated with different concentrations of metformin (DU145: 0, 50, 100, 150, and 200 mM and PC3: 0, 12.5, 50, 100, and 150 mM) and observed for 24 h. As shown in Supplementary Figure 2, fewer cells survived when treated with 100 mM of metformin at 24 h. The CCK8 assay showed that cell viability was significantly reduced when treated with 100 mM of metformin (*P* < 0.01) (Figure 1B). Then the control cells (DU145-NC) were treated with 60 mM of metformin. Cells transfected with the lentiSAMv2 pooled library were treated with 100 mM of metformin for 24 h (Figure 1C).

### Enriched sgRNAs in DU145 Cells With Metformin Resistance

**Figure 2:**
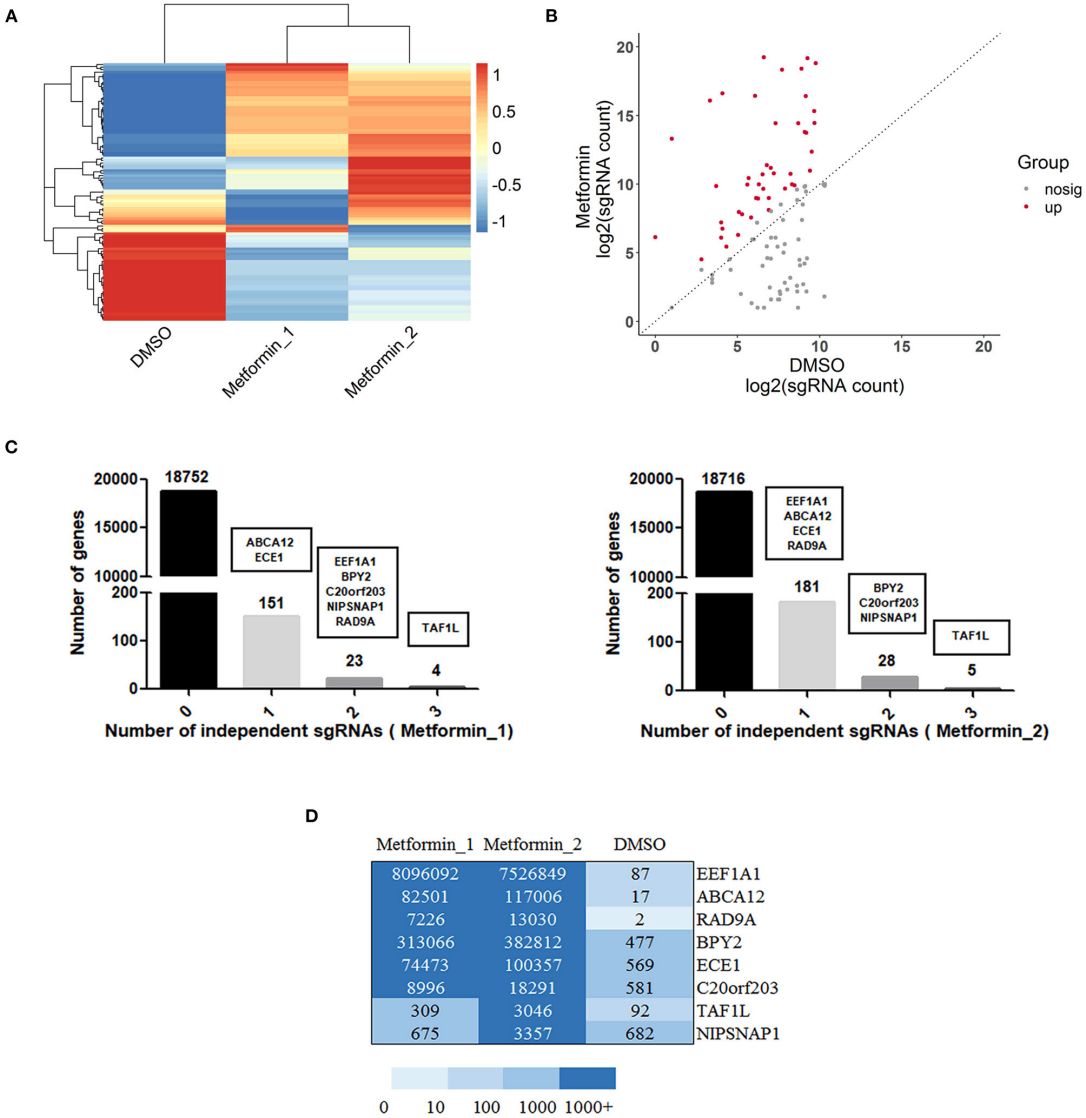
Gain of function screening in DU145 cells reveals genes that confer metformin resistance. **(A)** A heatmap displaying median-centered counts for differentially abundant sgRNAs; **(B)** scatter plot showing the significantly upregulated sgRNA enriched after metformin treatment; **(C)** number of genes with 0, 1, 2, or 3 significantly enriched resistant sgRNAs. Genes were selected by reads of sgRNA and sgRNA diversity, as shown in black boxes. **(D)** A heatmap displaying the resistant sgRNAs counts of the selected eight genes.

With the use of sgRNAs to target each gene of metformin-resistant DU145 cells after metformin treatment, a set of differentially upregulated sgRNAs (representing different particular genes) was enriched, which can be seen in the cluster heat map and scatter plot (Figures 2A,B), suggesting the gain of these particular genes contributes to metformin resistance. Furthermore, we screened the genes based on the total reads of sgRNA and sgRNA diversity for subsequent validation. As shown in Figure 2C, genes that have three significantly sgRNAs were detected in DU145 cells with metformin resistance. There were 23 or 28 genes that appeared to have two resistant sgRNAs, and 151 or 181 genes with one resistant sgRNA. Collectively, EEF1A1, BPY2, ABCA12, ECE1, TAF1L, C20orf203, NIPSNAP1, and RAD9A were the genes ultimately selected for further study (Figure 2D).

### Overexpression of Candidate Genes in PCa Cells Are Resistant to Metformin

To verify whether these candidate genes which were identified from the CRISPRa screening were resistant to metformin, we generated individual gene overexpression in the DU145 and PC3 cells. Compared with the NC group, each candidate gene individually overexpressed by CRISPRa was highly expressed in PCa cells (Supplementary Figure 3). We then tested their susceptibility to metformin with CCK8 assays.

**Figure 3:**
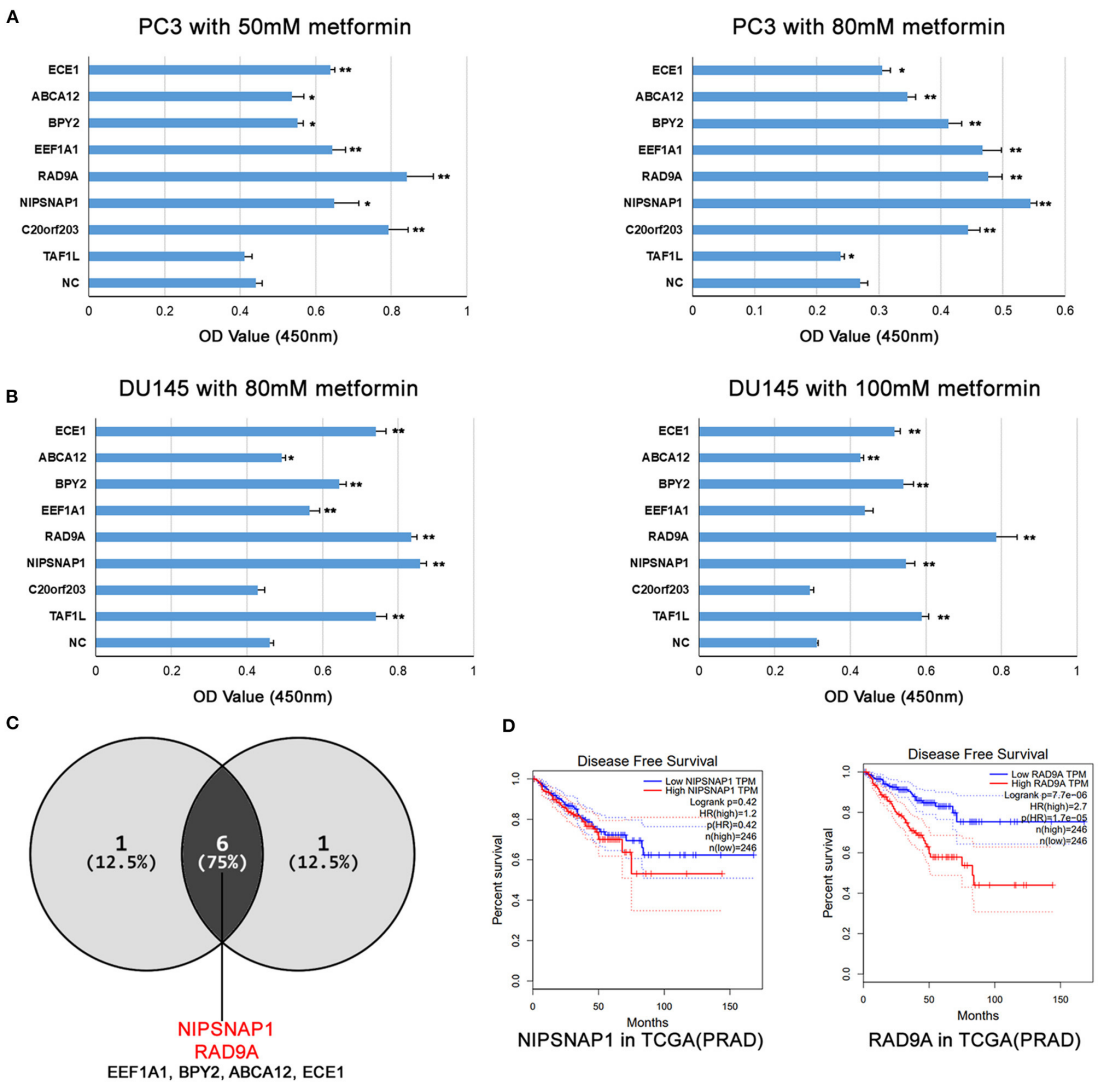
The overexpression of the candidate genes increases metformin resistance in PCa cell lines. **(A,B)** PC3 and DU145 cell lines with single gene overexpression were treated with the indicated doses of metformin for 24 h, and cell viability was measured using a CCK8 assay. Data were represented as mean ± SEM of a representative experiment. **(C)** The Venn diagram shows that the candidate genes increases metformin resistance in PC3 and DU145. **(D)** Disease Free Survival analysis of RAD9A and NIPSNAP1 in TCGA-PRAD dataset.

As shown in Figure 3A, overexpression of ECE1, ABCA12, BPY2, EEF1A1, RAD9A, NIPSNAP1, and C20orf203 exhibited significant pro-proliferative effects on PC3 cells compared with the NC group. Similar results were also obtained in DU145 cells. When treated with 80 and 100 mM of metformin, DU145 cells overexpressing ECE1, ABCA12, BPY2, EEF1A1, RAD9A, NIPSNAP1, and TAF1L grew faster than that of the control group (Figure 3B). These data show that the overexpression of these candidate genes (Figure 3C; ECE1, ABCA12, BPY2, EEF1A1, RAD9A, NIPSNAP1) in metformin-treated PCa cells increased cell survival. Interestingly, RAD9A and NIPSNAP1 were the top two increased groups in both cell lines. Based on the TCGA-PRAD dataset, a high level of RAD9A was correlated with poor prognosis while NIPSNAP1 showed no effect on the prognosis of PCa patients (Figure 3D).

### Functional Enrichment in the mRNA Level of RAD9A in Prostate Cancer

**Figure 4:**
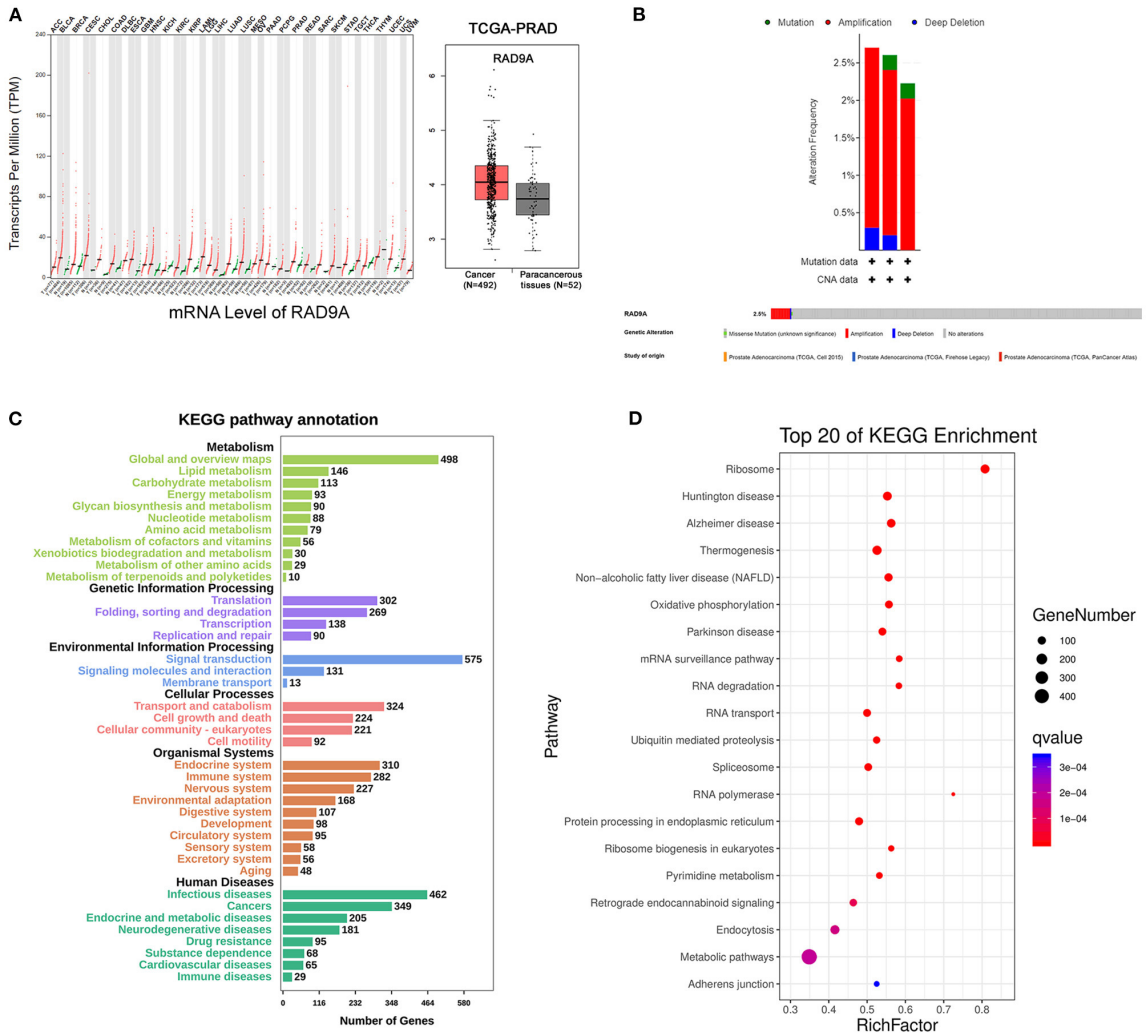
RAD9A gene expression and mutation analysis in prostate cancer. **(A)** The scatter of RAD9A expression levels in cancers from GEPIA (left), the boxplot of RAD9A expression levels in prostate cancer from TCGA (right). **(B)** Alteration frequency and genetic alteration analysis in prostate cancer by cBioPortal. **(C,D)** The top 20 pathways of KEGG pathway analysis, the input genes are related with RAD9A expression (*Q*-value < 0.01, *r* > 0.3, or *r* < −0.3) from the TCGA dataset. The size of the bubble represents the gene number, and the color indicates the *Q*-value.

To further investigate the role of RAD9A at the mRNA level, some bioinformatic analysis was conducted. The results of GEPIA revealed that the RAD9A expression levels were significantly upregulated in the vast majority of cancers including prostate cancer (Figure 4A). The cBioPortal showed that the percentage of RAD9A genetic alterations including mutation, amplification, and deep depletion were 2.7% (9/333), 2.61% (13/499), and 2.23% (12/494), respectively (Figure 4B). Figures 4C,D shows the number of KEGG pathway annotations in diverse categories and the top 20 KEGG pathways. The results showed that KEGG pathways were enriched mainly in cancers of human diseases, and the endocrine and immune system of organismal systems. In addition, metabolic pathways, mRNA surveillance pathway, and endocytosis were enriched in the top 20 KEGG pathways that might play indispensable roles in prostate cancer.

### Functional Enrichment in the Protein Level of RAD9A

**Figure 5:**
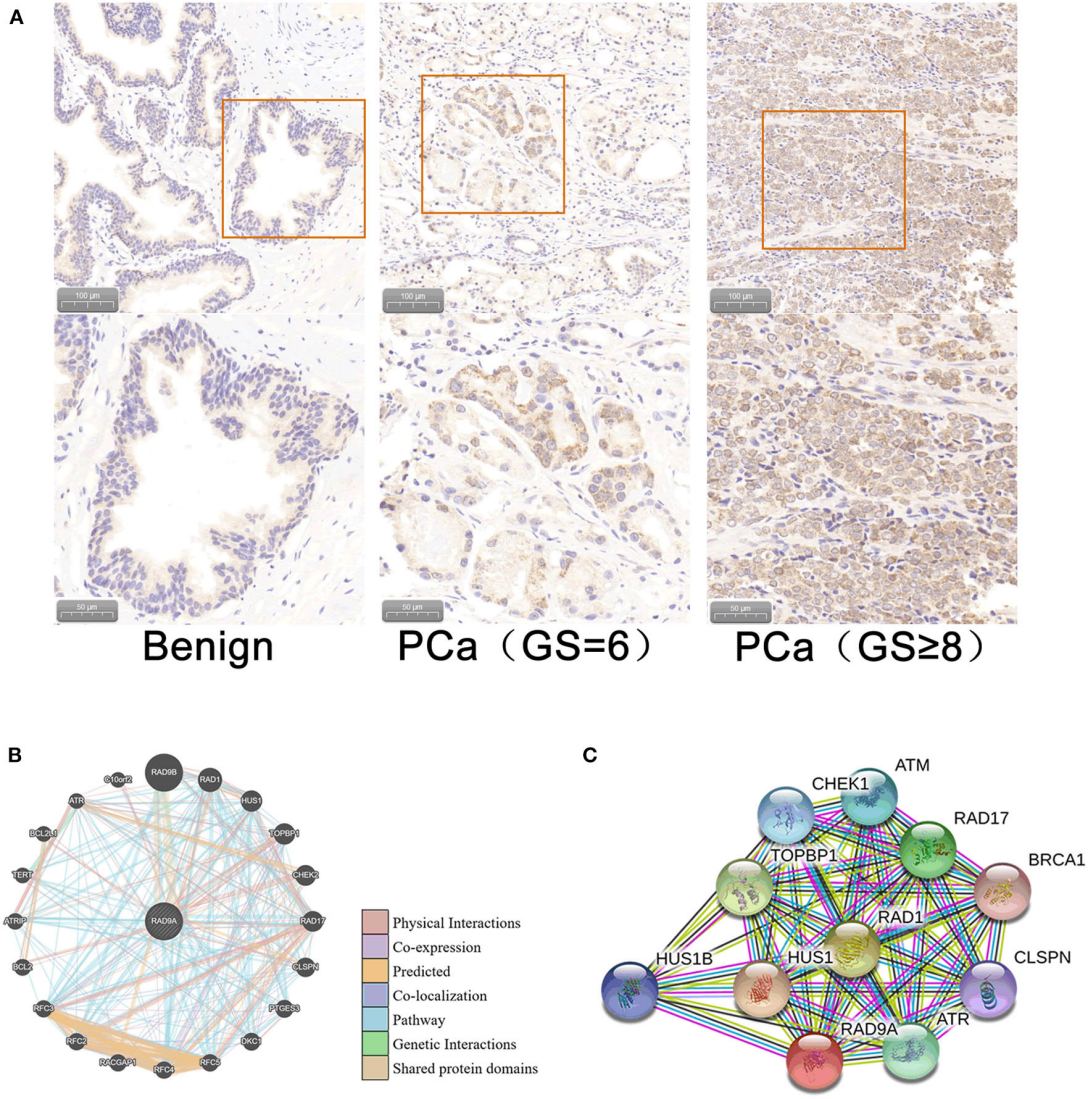
Cellular sub-localization and enrichment analysis of RAD9A at the protein level. **(A)** Immunohistochemistry analysis of the RAD9A antibody in a PCa tissue microarray. **(B)** The network for RAD9A and the 20 most frequently altered neighboring genes in the GeneMANIA dataset. **(C)** Protein-protein interaction network for RAD9A in the STRING dataset.

Immunohistochemistry analysis of the RAD9A antibody was undertaken in a PCa tissue microarray and was found to be mainly located in the nuclear and cytoplasm (Figure 5A). The positive staining areas of RAD9A had a significantly stronger intensity than those in benign tissues (*P* < 0.001; IRS of cancerous tissue: 5.52 ± 1.31; IRS of benign tissue: 2.31 ± 1.12; Table 2). Moreover, PCa tissues samples that had high RAD9A protein expression levels were more likely to have high Gleason scores (*P* < 0.001). Besides, we explored the network for RAD9A and the 20 most frequently altered neighbor genes using GeneMANIA (Figure 5B) and identified the interactions of the RAD9A protein expression level by using STRING (Figure 5C). The GeneMANIA analysis found physical interactions of RAD9A with BCL2, BCL2L1, C10orf2, TOPBP1, HEK2, CLSPN, and RAD17, the shared protein domain of RAD9A with RAD9B, and STRING analysis showed that RAD9A interacted with ATM, RAD1, RAD17, BRCA1, CLSPN, ATR, HUS1, HUS1B, TOPBP1, CHEK1, and ATM.

**Table 2:** RAD9A expression level with clinical features in prostate cancer TMA.

**Clinical features**	**TMA**		
	***N***	**Mean ± *SD***	***P***
**TYPE OF TISSUE**			
Benign	10	2.31 ± 1.12	<0.001
Malignant	70	5.52 ± 1.31	
**GLEASON SCORE**			
6	14	4.13 ± 0.32	<0.001
7	12	5.38 ± 0.82	
≥8	46	6.35 ± 0.79	
**PATHOLOGICAL STAGE**			
≤T2c	40	5.14 ± 0.22	0.921
T3a-T4	16	5.28 ± 0.47	

### Immune Cell Infiltration Analysis

**Figure 6:**
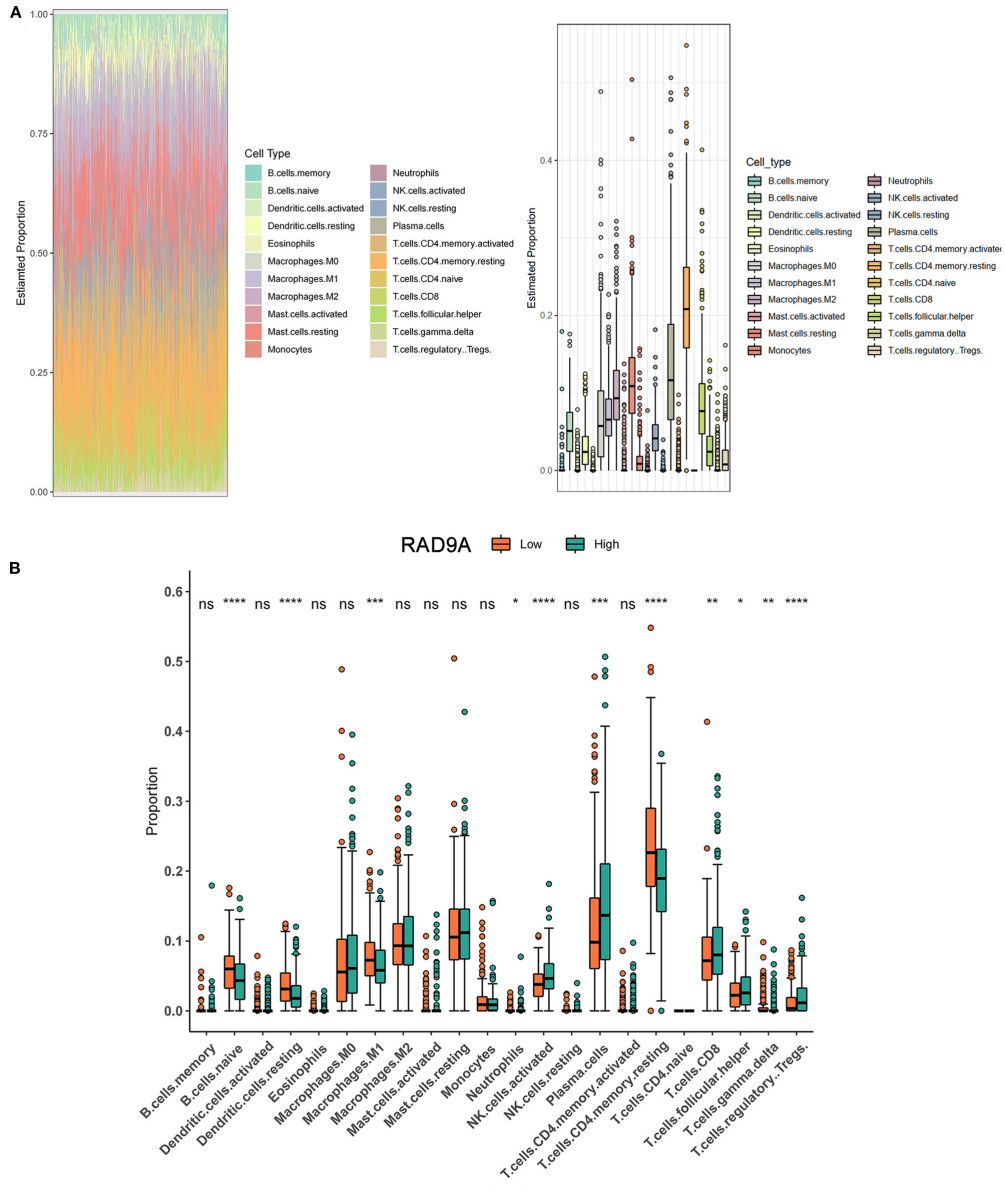
Intratumoral immune cell composition analysis. **(A)** The proportion of 22 immune cell types in prostate cancer from TCGA datasets. **(B)** The boxplot shows the different infiltration levels of 22 immune cell types between the high and low RAD9A expressions. *P < 0.05; **P < 0.01; ***P < 0.001. ****P < 0.0001.

The proportion of 22 immune cell types in PCa from the TCGA dataset showed that some cells were highly abundant, such as resting CD4 memory T cells, plasma cells, and resting mast cells (Figure 6A). The infiltration levels of immune cells between low and high RAD9A expression groups revealed that a high expression of RAD9A was consistent with a high proportion of regulatory T cells, T follicular helper cells, CD8+ T cells, plasma cells, activated NK cells (*P* < 0.05), and that RAD9A was negatively related to the infiltrating levels of naive B cells, resting dendritic cells, M1 macrophages, and resting memory CD4 T cells (Figure 6B).

## Discussion

Metformin, 1, 1-dimethylbiguanide hydrochloride, has been proven to be effective against a variety of tumors, including colon, lung, breast, endometrial, pancreatic, and prostate cancer (Gong et al., 2006; Comstock et al., 2007; Boorjian et al., 2012; Wahdan-Alaswad et al., 2016; Duan et al., 2017; Tong et al., 2017). Accumulating evidence suggests that the metformin anti-tumor effect is multifaceted (Pineda et al., 2015; Daugan et al., 2016). First, metformin can block the PI3K/MAPK pathway in cell growth to decrease glycaemia and insulinemia (Chen et al., 2009; Gallagher and LeRoith, 2011; Weinberg and Chandel, 2015). Second, metformin can activate the AMPK pathway to influence tumor metabolism, inflammation, and angiogenesis (Choi and Park, 2013; Mohammed et al., 2013). As a potential anticancer drug, metformin has become a research hotspot. However, patients with diseases including cancer will eventually become drug resistant after long-term use of metformin, and the underlying mechanisms involved in resistance to metformin is still unclear. In this study, genome-scale CRISPRa screening and deep sequencing analysis were applied to find novel genes involved in metformin resistance.

In our CRISPRa screening and deep sequencing analysis, EEF1A1, BPY2, ABCA12, ECE1, TAF1L, C20orf203, NIPSNAP1, and RAD9A were the potential metformin resistance genes. Furthermore, single gene overexpression in DU145 and PC3 cell assays ruled out TAF1L and C20orf203. What is more, RAD9A and NIPSNAP1 were in the top two increased groups and a high level of RAD9A correlated with a smaller disease-free possibility. From the above results, we chose RAD9A for further analysis.

RAD9 checkpoint clamp component A (RAD9A) is a kind of cell cycle checkpoint protein (Sierant et al., 2010). Due to its 3' to 5' exonuclease activity, RAD9A excels at sensing DNA dual-strand breaks, responsively forming a heterotrimeric ring-shaped complex involving RAD9A, RAD1, and HUS1, which activates the CHK1 checkpoint kinase and initiates DNA damage repair (DDR) during the G2/M cell cycle period (Greer et al., 2010; Balmus et al., 2016; Sierant and Davey, 2018). Recently, RAD9A has been proven to play a vital role in cancer proliferation, metastasis, and drug sensitivity (Balmus et al., 2016; Broustas et al., 2019). It is reported that knockdown of RAD9A enhanced esophageal cancer sensitivity to trichostatin A inducing DNA damage (Pang et al., 2016). More importantly, apart from functioning as a part of the RAD9A-HUS1-RAD1 complex, RAD9A independently drives prostate cancer metastasis by controlling AGR2 abundance (Broustas et al., 2019). These findings indicate that RAD9A may act as a key regulator in modulating metformin resistance in prostate cancer, which is required for further research. Nonetheless, the role of RAD9A in PCa drug resistance is still unknown.

To ascertain the function of RAD9A in PCa, we systematically performed some bioinformatic analyses. At the mRNA level, RAD9A were significantly upregulated in many cancers including prostate cancer. Besides, we enriched cancers, metabolic pathways, and the mRNA surveillance pathway in KEGG pathways analysis. Based on our PCa TMA, RAD9A was mainly located in the nuclear and cytoplasm. The positive staining areas of RAD9A had a significantly stronger intensity in cancer tissues and were in the higher Gleason scores group. Additionally, RAD9A potentially interacted with some famous oncogenes, such as BRCA1, CLSPN, and BCL2 based on GeneMANIA and STRING. These analyses suggested that RAD9A may have an important oncogene effect on PCa. However, more experimental evidence is needed.

Growing evidence has shown that metformin exhibits anti-tumor capability *via* adjusting the tumor immune microenvironment (TIME). Wang et al. (2020) reported that metformin reprogrammed the TIME by increasing infiltrated CD8+ cytotoxic T lymphocyte, CD20+ B lymphocyte, tumor-suppressive (CD11+), and decreasing tumor-promoting (CD163+) macrophages. At the same time, metformin triggered AMPK, STAT3 inactivation, and altered cytokine production in the immune cells. The above effects occurred in a more anti-tumoral state which may be beneficial to immunotherapy. Based on the metformin resistance effect of RAD9A, we wondered whether RAD9A would counteract metformin in TIME. Therefore, we explored the role of RAD9A in the TIME by using the TCGA-PRAD dataset as the background data. In our CIBERSORT results, regulatory T cells, T follicular helper cells, CD8+ T cells, plasma cells, and activated NK cells were correlated with a high RAD9A level. Besides, naive B cells, resting dendritic cells, M1 macrophages, and resting memory CD4 T cells were correlated with a low RAD9A level. RAD9A showed a tumor promoting effect in our data. Nevertheless, it was unexpected that CD8+ T cells and activated NK cells, two players which act in an anti-tumor role in TIME (Gajewski et al., 2013), were positively correlated with RAD9A. Because of the complexity of TIME, it is hard to explain this with a single cause. We believe that the regulatory T cells which are upregulated in the high RAD9A level group occupy an important position in the inhibition of CD8+ T cells and activated NK cells (Terme et al., 2008). From the above analyses, we presumed that RAD9A could counterbalance the “more anti-tumoral state” formed by metformin.

In summary, our data provide some new evidence for ascertaining the genetic determinants of metformin resistance in DU145 cell using the CRISPR-Cas9 genome-wide screening strategy. We demonstrated that ECE1, ABCA12, BPY2, EEF1A1, RAD9A, and NIPSNAP1, contribute to *in vitro* resistance to metformin in PCa cells. Furthermore, RAD9A may be involved in TIME adjustment of metformin by upregulating regulatory T cells. These findings suggested that the differentially resistant genes of metformin detected by our approach could therefore be of great importance in identifying novel options for PCa therapy. However, further experiments should be performed to validate the underlying mechanisms of RAD9A and other candidate genes in metformin resistance.

## Ethics Statement

The studies involving human participants were reviewed and approved by Huizhou Municipal Central Hospital. The patients/participants provided their written informed consent to participate in this study.

## Author Contributions

JC, YH, and WZ designed the research, analyzed the data, and wrote the paper. ZT, HZ, and ML designed the figures and tables of the manuscript. JC wrote the first draft of the manuscript. XY and WZ completed the second draft of this paper. SF and JL performed the literature review. All contributors reviewed and edited the manuscript and unanimously approved its final version.

## Conflict of Interest

The authors declare that the research was conducted in the absence of any commercial or financial relationships that could be construed as a potential conflict of interest.

## Supplementary Material

Supplementary Figure 1 PCR amplification of sgRNA region. Successful transfection of the lentiSAMv2 vector in DU145 cells, as indicated by electrophoresis of the PCR amplification region (209 bp). M: 2000 bp DNA marker; 1: PCR amplification products of DU145 cells; 2: PCR amplification products of lentiSAMv2 control plasmid transfected with DU145 cells; 3: PCR amplification products of CRISPR/Cas9 SAM pooled library plasmid transfected with DU145 cells.

Supplementary Figure 2 Optical microscopic images of PCa cells treated with metformin (DU145: 0, 50, 100,150, 200 mM and PC3: 0, 12.5, 50, 100, 150 mM).

Supplementary Figure 3 Quantitative polymerase chain reaction (qPCR) assays showing the overexpression levels of EEF1A1, BPY2, ABCA12, ECE1, TAF1L, C20orf203, NIPSNAP1, and RAD9A mRNA in PCa cells.
