# Supplementary figures and images for: Genome-Scale CRISPR-Cas9 Transcriptional Activation Screening in Metformin Resistance Related Gene of Prostate Cancer
Source: Front Cell Dev Biol. 2021 Jan 26;8:616332. doi: 10.3389/fcell.2020.616332 (PMC7870801; doi:10.3389/fcell.2020.616332)

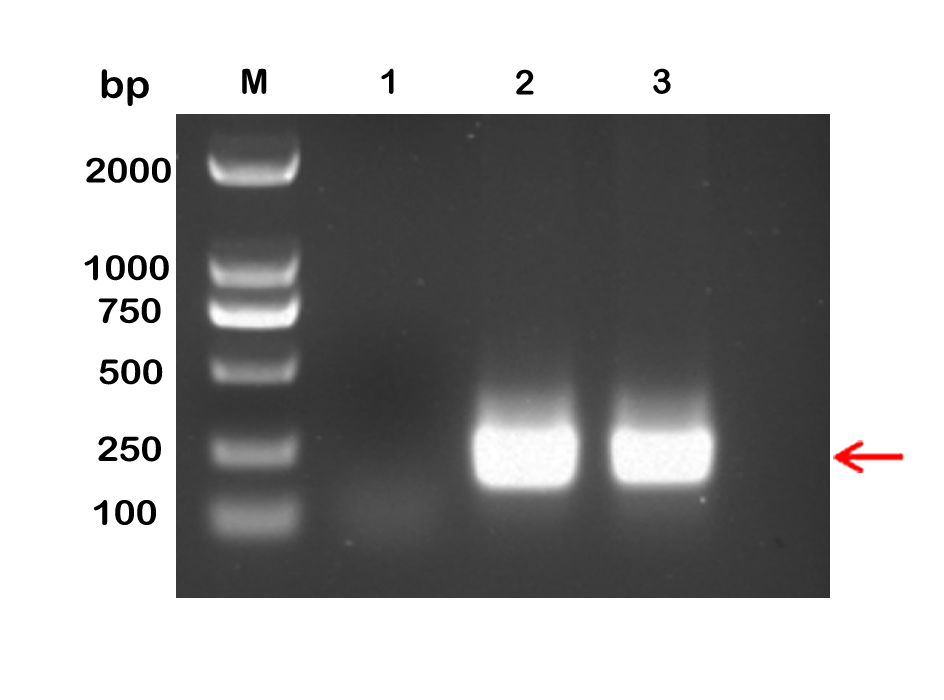

Supplement: Supplementary Figure 1 — PCR amplification of sgRNA region. Successful transfection of the lentiSAMv2 vector in DU145 cells, as indicated by electrophoresis of the PCR amplification region (209 bp). M: 2000 bp DNA marker; 1: PCR amplification products of DU145 cells; 2: PCR amplification products of lentiSAMv2 control plasmid transfected with DU145 cells; 3: PCR amplification products of CRISPR/Cas9 SAM pooled library plasmid transfected with DU145 cells. [file Image_1.png]

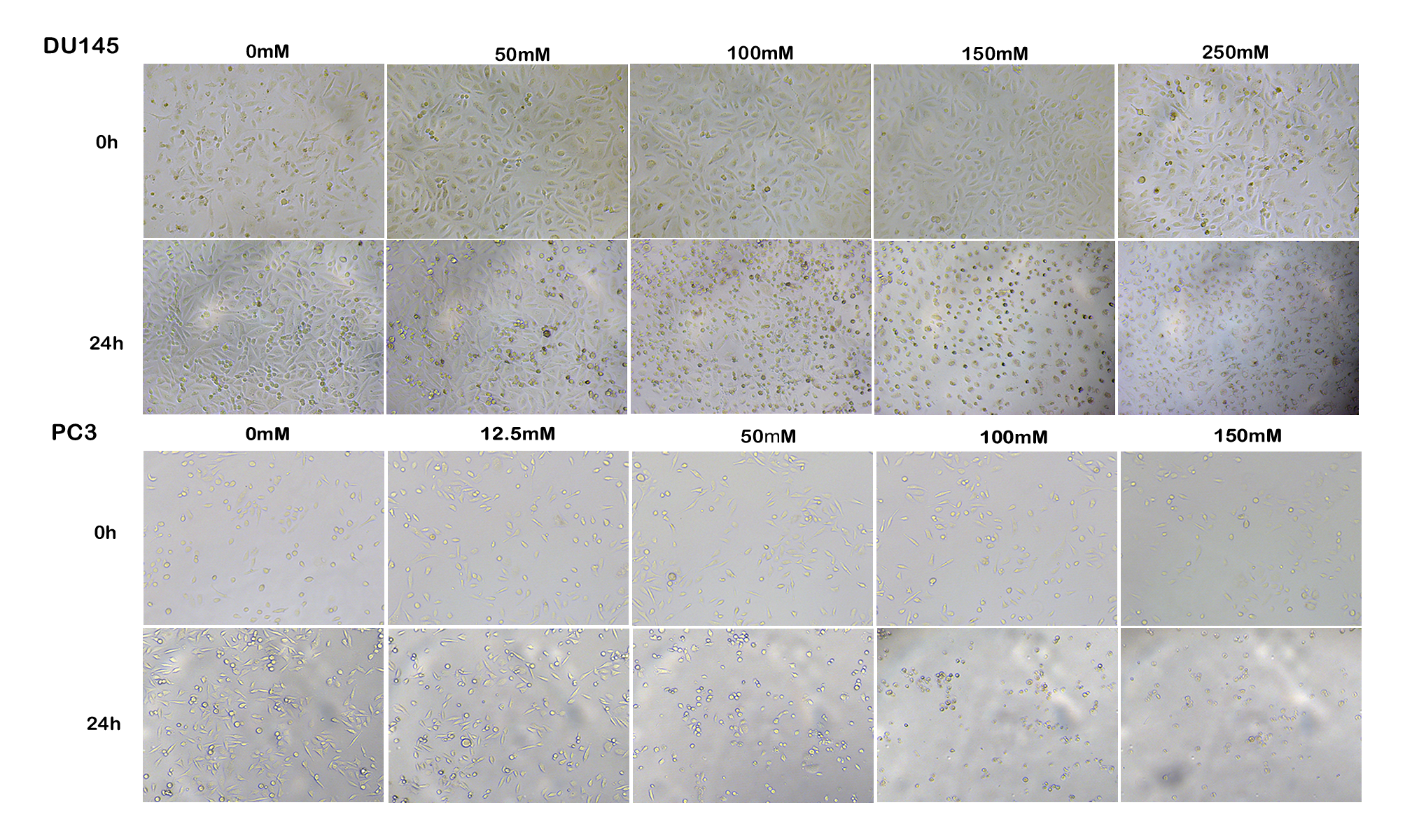

Supplement: Supplementary Figure 2 — Optical microscopic images of PCa cells treated with metformin (DU145: 0, 50, 100,150, 200 mM and PC3: 0, 12.5, 50, 100, 150 mM). [file Image_2.png]

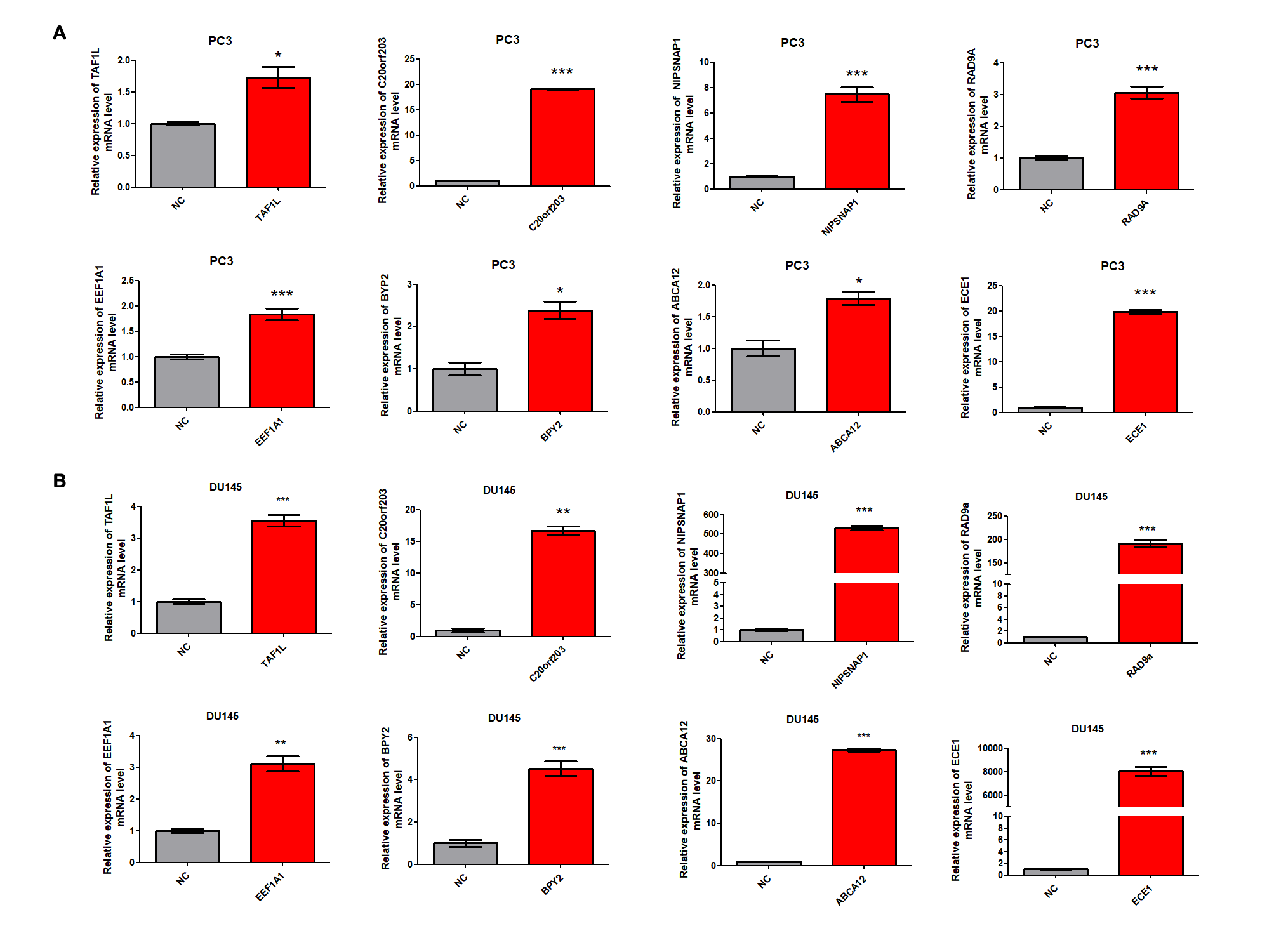

Supplement: Supplementary Figure 3 — Quantitative polymerase chain reaction (qPCR) assays showing the overexpression levels of EEF1A1, BPY2, ABCA12, ECE1, TAF1L, C20orf203, NIPSNAP1, and RAD9A mRNA in PCa cells. [file Image_3.png]
